# A full pipeline of diagnosis and prognosis the risk of chronic diseases using deep learning and Shapley values: The Ravansar county anthropometric cohort study

**DOI:** 10.1371/journal.pone.0262701

**Published:** 2022-01-20

**Authors:** Habib Jafari, Shamarina Shohaimi, Nader Salari, Ali Akbar Kiaei, Farid Najafi, Soleiman Khazaei, Mehrdad Niaparast, Anita Abdollahi, Masoud Mohammadi

**Affiliations:** 1 Department of Statistics, Razi University, Kermanshah, Iran; 2 Department of Biology, Faculty of Science, Universiti Putra Malaysia, Serdang, Selangor, Malaysia; 3 Department of Biostatistics, School of Health, Kermanshah University of Medical Sciences, Kermanshah, Iran; 4 Sleep Disorders Research Center, Kermanshah University of Medical Sciences, Kermanshah, Iran; 5 Department of Computer Science, Sharif University of Technology, Tehran, Iran; 6 Research Center for Environmental Determinants of Health, School of Public Health, Kermanshah University of Medical Sciences, Kermanshah, Iran; 7 Cellular and Molecular Research Center, Gerash University of Medical Sciences, Gerash, Iran; Indiana University Purdue University at Indianapolis, UNITED STATES

## Abstract

Anthropometry is a Greek word that consists of the two words “Anthropo” meaning human species and “metery” meaning measurement. It is a science that deals with the size of the body including the dimensions of different parts, the field of motion and the strength of the muscles of the body. Specific individual dimensions such as heights, widths, depths, distances, environments and curvatures are usually measured. In this article, we investigate the anthropometric characteristics of patients with chronic diseases (diabetes, hypertension, cardiovascular disease, heart attacks and strokes) and find the factors affecting these diseases and the extent of the impact of each to make the necessary planning. We have focused on cohort studies for 10047 qualified participants from Ravansar County. Machine learning provides opportunities to improve discrimination through the analysis of complex interactions between broad variables. Among the chronic diseases in this cohort study, we have used three deep neural network models for diagnosis and prognosis of the risk of type 2 diabetes mellitus (T2DM) as a case study. Usually in Artificial Intelligence for medicine tasks, Imbalanced data is an important issue in learning and ignoring that leads to false evaluation results. Also, the accuracy evaluation criterion was not appropriate for this task, because a simple model that is labeling all samples negatively has high accuracy. So, the evaluation criteria of precession, recall, AUC, and AUPRC were considered. Then, the importance of variables in general was examined to determine which features are more important in the risk of T2DM. Finally, personality feature was added, in which individual feature importance was examined. Performing by Shapley Values, the model is tuned for each patient so that it can be used for prognosis of T2DM risk for that patient. In this paper, we have focused and implemented a full pipeline of Data Creation, Data Preprocessing, Handling Imbalanced Data, Deep Learning model, true Evaluation method, Feature Importance and Individual Feature Importance. Through the results, the pipeline demonstrated competence in improving the Diagnosis and Prognosis the risk of T2DM with personalization capability.

## 1. Introduction

Chronic diseases (such as diabetes, hypertension, cardiovascular disease, stroke and heart) due to their long duration and sometimes incurable will always be with the patient until death and this leads to internal and External abnormalities for the sick person and his/her family and relatives [1]. Accordingly, both the person and the family and ultimately the community and the system must provide facilities to deal with these types of diseases and by staying with the patient to prevent more suffering of the patient and imposed costs to society [1]. So, identifying the type of chronic diseases and the factors affecting them and finally, finding a suitable model to prevent their occurrence can be useful [1, 2]. Accordingly, to rid society of the problems of these diseases, it seems that finding the effective factors and controlling them can be a long and useful step to prevent the growth of such diseases in society [2].

Anthropometric features such as height, weight, waist circumference, hip circumference and body mass index can be considered as factors that may cause chronic diseases and the abnormality of each of them can play a role in causing of chronic diseases. It should be noted that the occurrence of problems in the physical condition of a person has a decisive role in his/her disease and so, we can identify the causes of such problems and prevent the occurrence of such problems or find ways to treat them [2, 3].

According to the American Statistics Center, the world population in 1750 was about 800 million, which reached to 6 billion in 1999 and the latest statistics indicate a population of nearly 8 billion. Also, countries need to use the latest technologies in the world to meet the needs and necessities of life of the people. Therefore, many people do not have much mobility to do their daily work and due to the busy (work) to earn their living needs, don’t spend a lot of time on other non-professional activities such as exercise and this causes physical problems in their body [1–3]. It should be noted that in addition to this, conditions such as large population and high demand for consumables such as food, has led suppliers to prepare enough fast foods in order to earn both more money and supply materials [1–3]. This issue, in addition to being busy and sedentary, has caused people problems such as weight gain and etc., for many people especially those over 40 years old [3]. They suffer from excessive weight gain and diseases such as high blood pressure and blood sugar, and sometimes due to lack of health facilities and adequate access to medical centers and lack of necessary knowledge are with them for the rest of their lives and always suffer from these diseases [3]. Accordingly, research on these types of diseases and their causative agents, although may not cause their eradication (for sufficient reasons), but can help health officials to control them [1–3].

In Iran, similar to the current plan, plans and articles have been published including the article [1] that studied cardiovascular disease and the sample size of 7806 people were extracted by random cluster sampling method and correlation methods and linear and logistic regression were used to analyze the data. The result of that indicates the relationship between systolic and diastolic blood pressure with weight. Also, a study on the relationship between weight and blood pressure has been conducted since 2000 with the support of Isfahan University of Medical Sciences [2], whose data source is Isfahan Healthy Heart data and The sample size is 12514 people that the age range is 38 years and Chi-square and analysis of variance were used for data analysis and the result shows the relationship between weight and blood pressure so that weight increases with increasing the age and causes high blood pressure. In 2013, an article was published [3] in which using a sample of 140 patients of Shariati and Shahid Rajaei hospitals in Karaj, the relationship between anthropometric indices and chronic cardiovascular disease were investigated by Statistical methods such as t-test, one-way analysis of variance and correlation coefficient for analyzing the data and the obtained results indicated the relationship between the two variables.

In addition to the above studies, also an article published in 2016 [4] that is the result of a study on 5811 students in Ahvaz city by classified random sampling method extraction with Equal allocation. In this study, correlation coefficient and regression statistical methods were used to analyze the data. The result shows the relationship between pressure and age, height and weight of students. There are other articles in this field in the literature [5–7].

This study was conducted to understand the relationship between chronic diseases and their causes in a more scientific way and also extracting the impact of these factors on chronic diseases in order to at least move towards controlling and limiting the mentioned diseases by setting priorities and focusing on them, Similar to the articles in [8–10]. So, the aim of this study is to investigate the anthropometric characteristics of patients with chronic diseases (diabetes, hypertension, cardiovascular disease, heart attack and stroke) and to find the factors affecting these diseases and the intensity of the impact of each for precautionary measures [11–13].

Many risk predictions are based on statistical knowledge to predict the risk of type 2 diabetes in individuals [14–17]. Unlike traditional methods, machine learning can learn nonlinear interactions from large amounts of data. Recent researches show that machine learning techniques can describe the characteristics of patients and thus identify the diagnosis and prognosis of high-risk patients. In a low number of articles, extensive research has been conducted on the use of machine learning to assess risk in the general population [18]. However, current methods have focused only on comparing the performance of prediction techniques with the fixed number of variables, and they have also been performed on a small population sample. Therefore, the aim of this study is (1) to apply deep learning algorithm to anthropometric features to predict the risk of chronic disease in an urban population in Iran, (2) feature importance, and (3) individual feature importance for a new patient.

## 2. Method

### 2.1. Ethics approval and consent to participate

The study was approved by the ethics committee of Kermanshah University of Medical Sciences. Written informed consent was obtained from all participants in the study.

**Fig 1:**
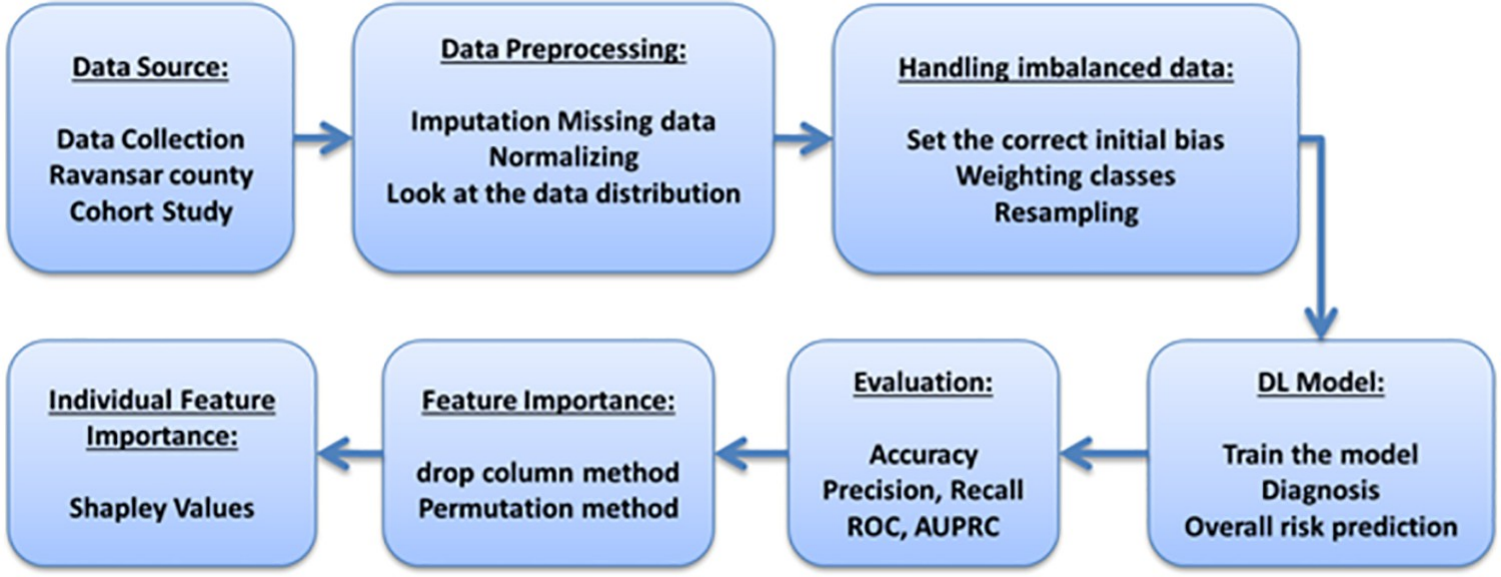
Methodology.

The general method of the processes performed in this research is shown in Fig 1. This process includes the following steps:

- Data source: collecting the data
- Data preprocessing: handling the missing values and outliers (Missed values are managed and very small)
- Handling imbalanced data: weighting each class based on majority
- DL model: designing and implementing a deep learning model
- Evaluation: measuring the performance of model
- Feature Importance: measuring the effect of each feature in general
- Individual Feature Importance: personalizing DL model for a new patient

### 2.2. Data source

This study is done descriptively-analytically. The research community is the people of Ravansar city that is one of the parts of Kermanshah province (Nr. 980879). Samples include people who refer to a specific treatment location at the request of the researcher and perform some tests and answer the questions posed by the researcher accurately. Data collection tools include questionnaires and interviews. The questionnaire required for this project is related to the questionnaire for measuring the anthropometric characteristics of individuals in the sample including five variables: height, weight, waist and hip circumference and body mass index, as well as data on chronic diseases of the sample members such as diabetes, blood pressure, cardiovascular disease, heart attack and stroke. This process will be done according to the sampling method and selecting the sample members and extracting information from them.

Among the participants with the age range of 34–70 years from Ravansar urban areas of Kermanshah (Iran), that were evaluated during the period time between March 2014 and April 2016, participants with the following characteristics were excluded from the study: 1) participants with incomplete information on variables, and 2 (participants who there was an error recording on their information. Finally, 10047 participants were considered for the present study.

### 2.3. Data preprocessing

**Fig 2:**
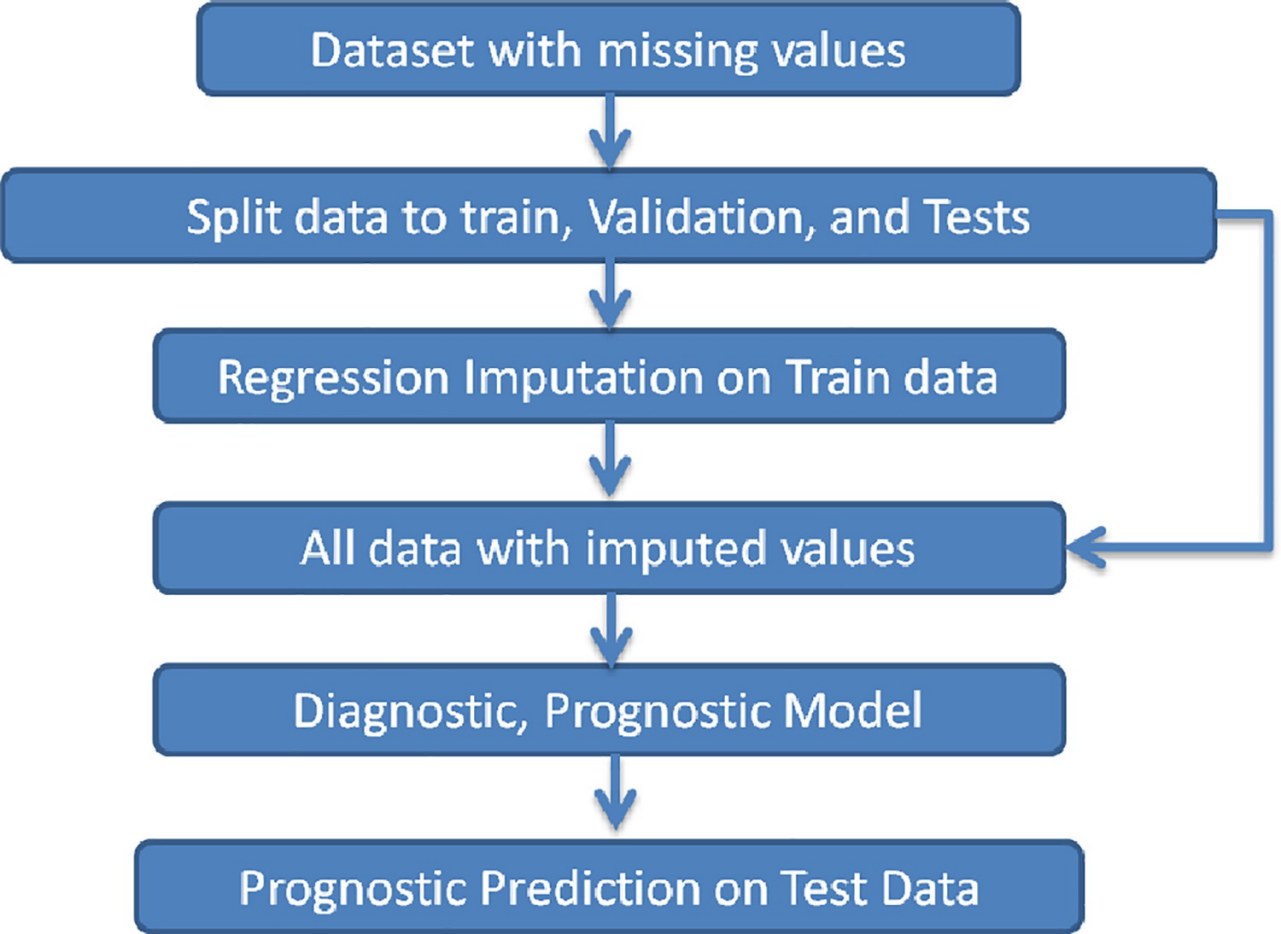
Impute the data to handle missing values.

The data collected for analysis has problems. One of them is missing values. These problems lead to a change in the distribution of data and confuse the learning algorithms for newcomers who do not follow this distribution. There are several ways to overcome this problem that two of the most important of them are as follows:

The first method is Mean Imputation, in which the average of training data is calculated for each feature column, and is replaced in all values of that column that have a Null value or are considered noise. In the second method i.e. Regression Imputation, a regression relationship is considered between the considered feature column and other input features for the training data. The value of outliers and Null data of this feature column is replaced by regression output value. We used the Regression imputation method to handle Null and outlier’s data. In this method, the data collection will have a better approximation of the data distribution. Fig 2 shows the process used in this study.

### 2.4. Handling imbalanced data

The data classes in this database are highly imbalance, in which the number of samples of the patient class is significantly less than the class data of healthy individuals. As a case study, in this study, we worked on the diagnosis of type 2 diabetes mellitus (T2DM) in which the number of patients with T2DM is 969 people against 10047 people in the whole society (9.64% of the total). We used Python programming language, and TensorFlow (version 2) framework to define class weights and to feed the model. In order to weight each class, first, we need to look at the data distribution of both classes. This has two advantages: First, we need to normalize input data to feed the model. Second, the data in a class may contain a much higher rate of extreme values.

### 2.5. Deep learning model architecture

**Fig 3:**
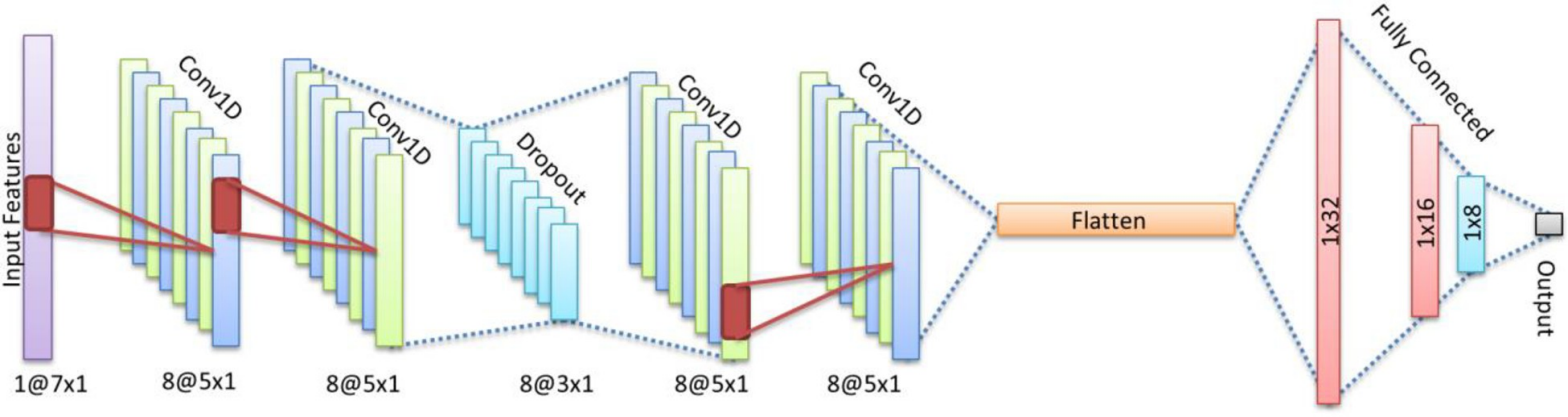
The architecture of deep learning method for diagnose T2DM.

In this study, we developed an algorithm to predict T2DM on the basis of a deep learning framework. This framework was used because it was able to combine conservation and generalization benefits with less feature engineering that is useful for analyzing of EHR data. This data set was divided into a train set (80%), a validation set (10%) and a test set (10%). The workflow for prediction the onset of T2DM using the deep model is shown in Fig 3 that patient characteristics are processed by a deep architecture. Deep neural network can better generalize new features by using compact embedding in small dimensions. In our framework, this component consists of convolution 1D, Dropout, Flatten, and Fully connected layers. To detect, we applied a binary output. Convolution layers contain Eight vectors (5 x 1) and dropout layers contain Eight vectors (3 x 1). The kernel size is 3x1 with zero-padding, and stride 1. After flattening, the fully connected layers are 32, 16, and 8. Finally, the binary output is learned by the network by the Sigmoid Activation function. Activation function in other layers was rectified linear unit (ReLU).

### 2.6. Evaluating the model

We used different criteria to evaluate the model such as Accuracy and Area under the Curve of the Precision-Recall Curve (AUPRC), Precision, Recall, Area Under Curve (AUC). The importance of diagnosing a person who is sick but the model mistakenly identifies him/her as a healthy person is much more than the error that a healthy person mistakenly diagnoses as sick. Therefore, in addition to Accuracy for model evaluation, we have considered the above criteria to determine the accuracy of the model. Accuracy is not a good metric for this task. For example, a classifier that identifies all people as healthy has an Accuracy of 90.36% (9.64%- 100%).

### 2.7. Feature importance

Today, the use of artificial intelligence, especially deep learning algorithms, is very common in both medical diagnosis and prognosis. The thing that is important in some diseases, such as tumors, Alzheimer, or diabetes, is to estimate the risk of the disease based on the current characteristics of the individual, which will lead to the prevention or early treatment of patients. Deep learning models generally have good accuracy in the diagnosis process (such as classification and segmentation). The thing that is important in prognosis is the personalization of the model for a new person that by which the model can give good accuracy from the occurrence time of an event such as a heart attack, According to the current characteristics of the person. From another point of view, this issue can be considered as the risk of a disease such as diabetes for the person in question. For example, blood pressure and age may be important factors for a heart attack. Generally, a model gives a fixed value to each of the characteristics of blood pressure and age that assesses the risk of a person to have a heart attack based on them. But, we know that the importance of these characteristics is very different for a young person with very high blood pressure and another person who is old and has normal blood pressure. Therefore, in this section we consider the case study of diabetes for the presented dataset, and after applying the deep learning model, we try to personalize the model for the new person. This is done by determining the importance of each attribute (whether for the data set in general, or for each individual).

### 2.8. Individual feature importance

In the previous section, we examined the general importance of features for our deep learning model of the entire data set. But, the important subject is that which features are more important for a new patient. Therefore, in this section, we obtain the importance of a feature in prediction of the disease for a particular person. We obtained the significance of each property in general for the model with methods such as the permutation method. But, in this section, it is more important to understand a single prediction of a patient than to understand a model.

## 3. Results

### 3.1. Data characteristics

General characteristics of the population under study have been presented in Table 1. The population includes 4764 males and 5283 females. Compared to participants without T2DM, individuals with T2DM tended to age older, had a larger BMI, and had higher waist circumference. Mean and standard deviation of anthropometric indexes and risk factors for chronic diseases have been presented in more detail in Table 1.

**Table 1:** Mean and standard deviation of anthropometric indexes and risk factors of chronic diseases.

	count	mean	Std.	min	25%	50%	75%	max
Age_At_Interview	10047	47.33	8.29	34	40	46	54	70
HeightCm	10047	162.77	9.3	114.4	155.4	162.1	170	197.7
WeightKg	10047	72.89	13.64	31.5	63.5	72.2	81.3	145.5
WaistCircumference	10047	97.34	10.51	57	90.5	97	104	148.5
HipCircumference	10047	102.63	8.87	64.7	97	102	107.6	150
BMI	10047	27.51	4.64	12.51	24.42	27.25	30.26	52.8
Age_T2DM	969	51.88	7.61	35	46	52	58	67
Age_Not_T2DM	9078	46.84	8.21	34	40	45.5	53	70
HeightCm_T2DM	969	160.96	9.09	137.6	153.9	160.2	167.8	185.7
HeightCm_Not_T2DM	9078	162.96	9.3	114.4	155.6	162.4	170.2	197.7
WeightKg_T2DM	969	73.3	12.7	37.5	64.8	72.8	81	127
WeightKg_Not_T2DM	9078	72.85	13.73	31.5	63.5	72.2	81.3	145.5
WaistCircumference_T2DM	969	99.65	9.89	65.2	93	99.5	106	132.5
WaistCircumference_Not_T2DM	9078	97.09	10.54	57	90	97	104	148.5
HipCircumference_T2DM	969	102.69	8.63	79.2	97	102	107.4	140.5
HipCircumference_Not_T2DM	9078	102.62	8.9	64.7	97	102	107.6	150
BMI_T2DM	969	28.29	4.33	15.59	25.37	28.09	30.79	48.84
BMI_Not_T2DM	9078	27.43	4.67	12.51	24.34	27.16	30.18	52.8

### 3.2. From raw data to evaluating DL model

After cleaning the data and applying regression imputation to handle missing values, the data is divided into three sections: train (80%), validation (10%), and test (10%). Validation set is used during network training to evaluate metrics although the model does not fit by it. The test set completely remains useless during training and is only used at the end to assess how much the model is generalized to new data. This is especially important when the database is imbalanced and the Lack of training data makes over fitting as a significant concern.

**Fig 4:**
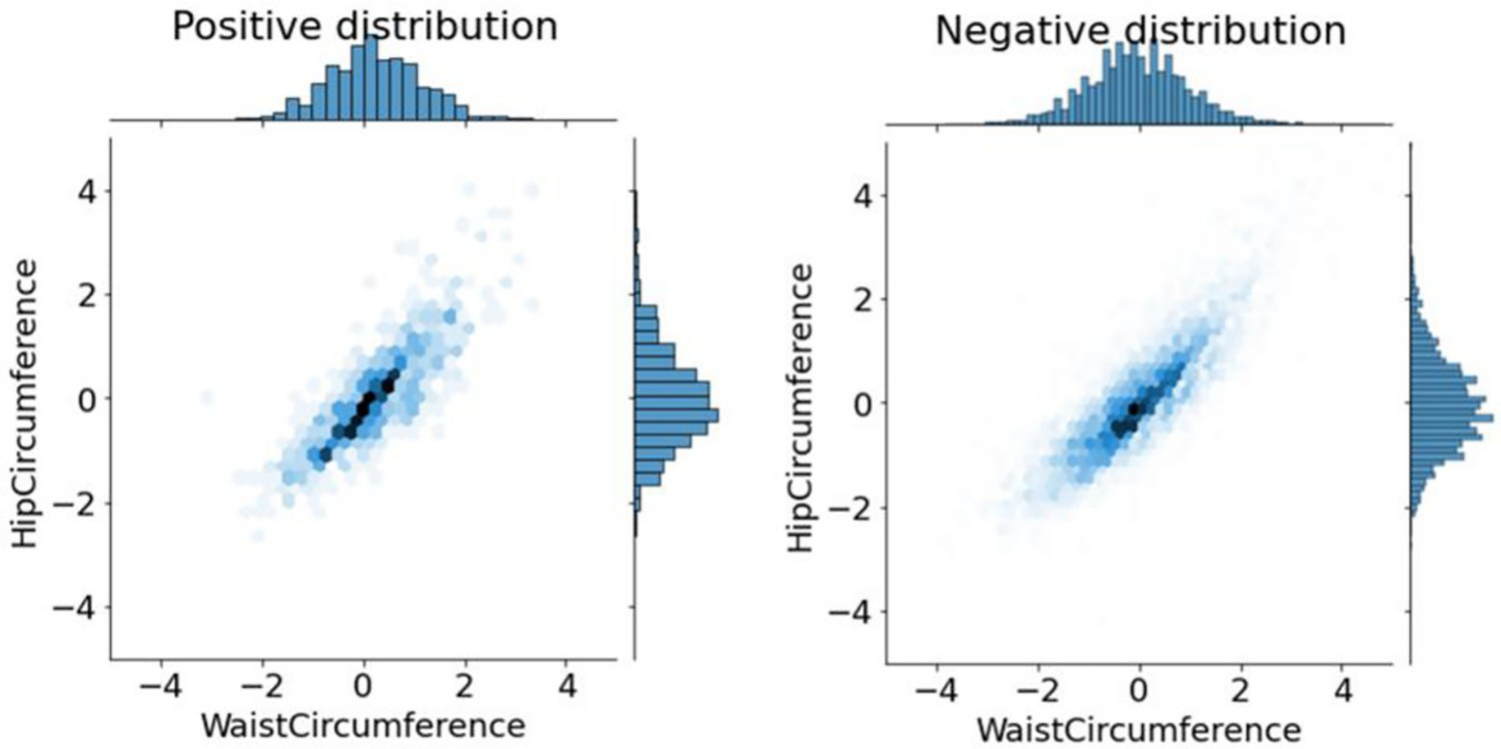
Looking at the data distribution for positive and negative classes.

After normalizing the train set, looking at the distribution of data on the positive and negative classes leads to observing the differences in the distributions. For example, Fig 4 shows this issue for waist and hip circumferences.

**Fig 5:**
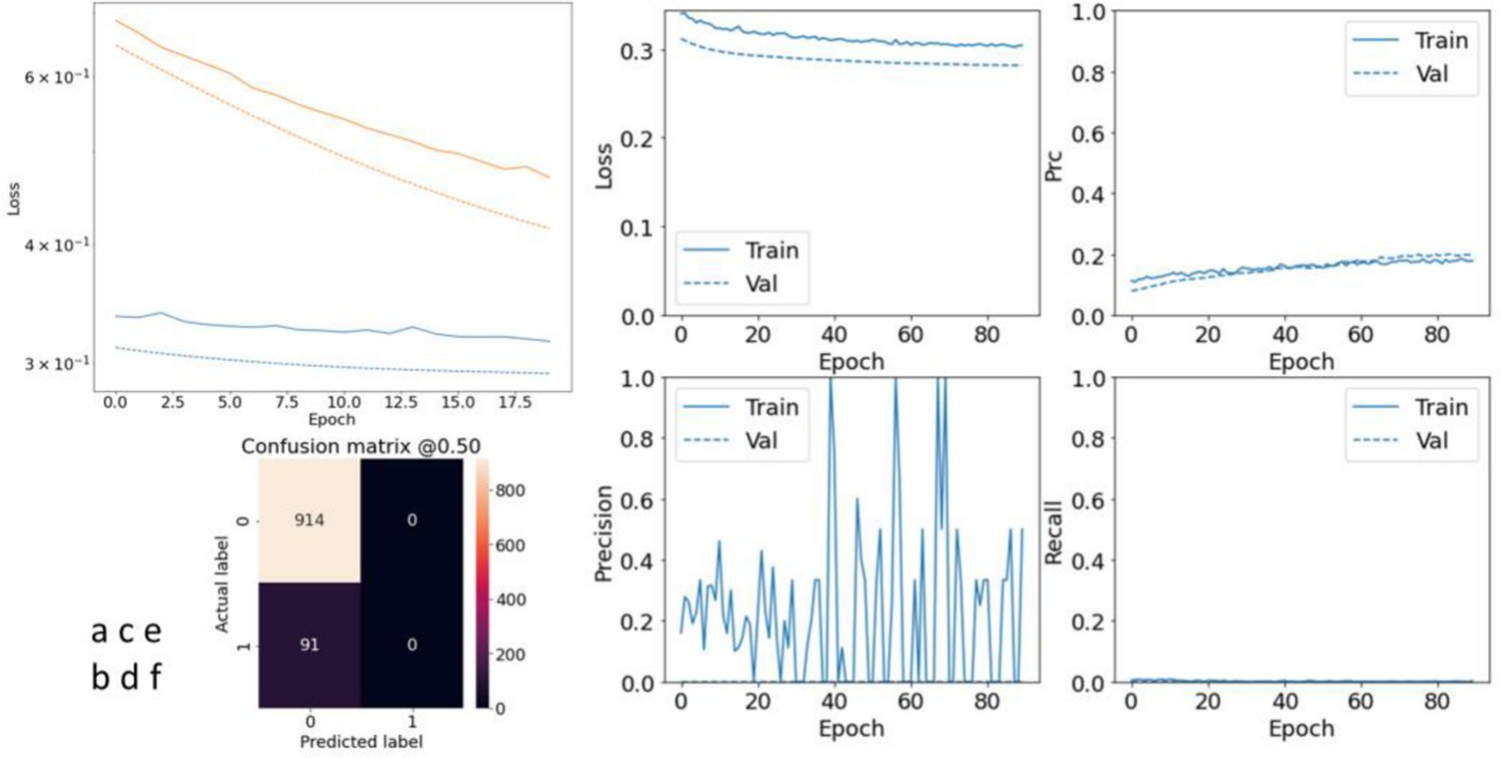
Evaluating baseline model performance. a) Advantage of bias initialization; b) confusion matrix; c-f) evaluate metrics.

The deep learning model used here has been shown in Fig 5, where the dropout layer is used to reduce over fitting and also, sigmoid function is located on the last layer to determine the Probability of disease. Although the initial bias is generally equal to log (2) = 0.69 ، it is better to calculate the bias for imbalanced data according to the probability of the presence of each of the classes. The probability of the presence of positive data is as follows:

$$p=\frac{pos}{pos+neg}=\frac{1}{1+e^{-b}}$$

Therefore, the value of b is calculated as follows:

$$b=-\log\left(\frac{1}{p}-1\right)=log(\frac{pos}{neg})$$

Therefore, the correct bias is $\log\left(\frac{969}{9078}\right)=-2.24$.

Considering this initialization, the loss value of this study which is binary cross entropy, is approximately equal to 0.3254 that is much smaller than the loss value in the case of considering naïve initialization. Following relationships shows the loss formula:

−plog(p)−(1−p)log(1−p)

**Fig 6:**
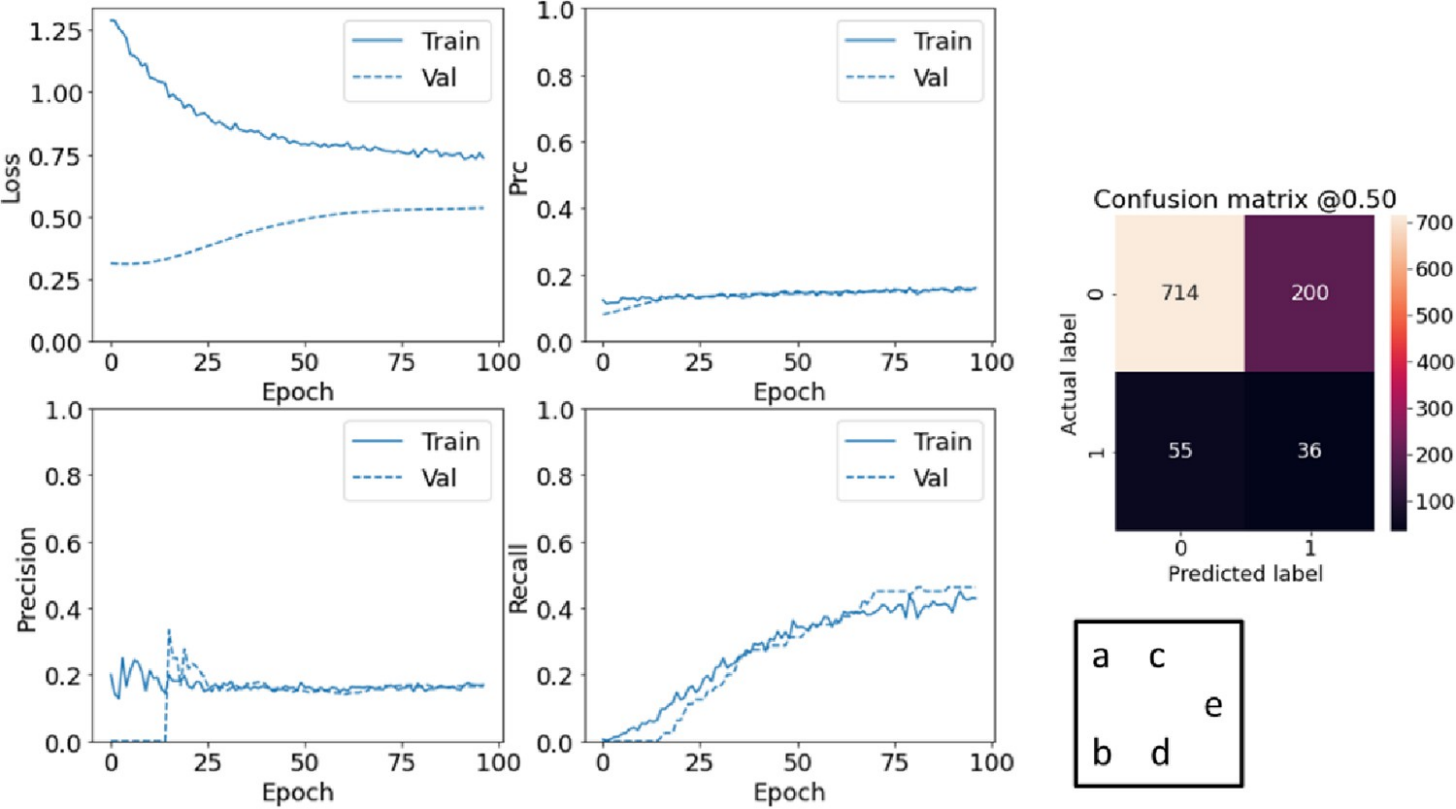
Evaluating weighted model performance. a-d) evaluate metrics; e) confusion matrix.

Fig 6 shows the improvement of the model performance in the case that initialization is done in the above form. The blue and orange lines are related to the loss in the cases with and without careful initialization, respectively. The ‘- -’ lines are for validation loss. The reason of better performance of the model in the validation set is inactivation of the dropout layer when evaluating the model. The confusion matrix in this figure shows that 91 patients are mistakenly known as healthy by the model. This error is very important in the T2DM task. Sections c-f in this figure shows other evaluations of this model. Ratings are low by both precision and recall, metrics that need to be increased for imbalanced data. However, classifiers generally have trouble in maximizing both of these metrics at the same time, especially when working on imbalanced data.

To overcome the problems of imbalanced data, two different techniques are used: Class weighting and Oversampling. In the first method, calculating the class weights helps to give a lot of weight to positive classes. This allows the deep network to pay more attention to positive class instances. The weight of each class is calculated by dividing the total number of data by the number of data in that class, which divides the total value by two to keep the loss to a similar magnitude. Therefore, the weight of the positive class was equal to $\frac{10047}{969*2}=5.18$ and the weight of the negative class was equal to 0.55. Because changing the weight of classes may affect the stability of the training due to the optimizer, an optimizer should be chosen whose step size does not depend on the size of the gradient. For this reason, Adam optimizer has been used in this research. Fig 6 shows that false positive values have increased. This reduces accuracy and precision. But, on the other hand, because we have more number of true positive, we expect an improvement in Recall and AUC metric. The task studied in this research focuses on the correct diagnosis of patients that the model performs much better than before in this regards.

**Fig 7:**
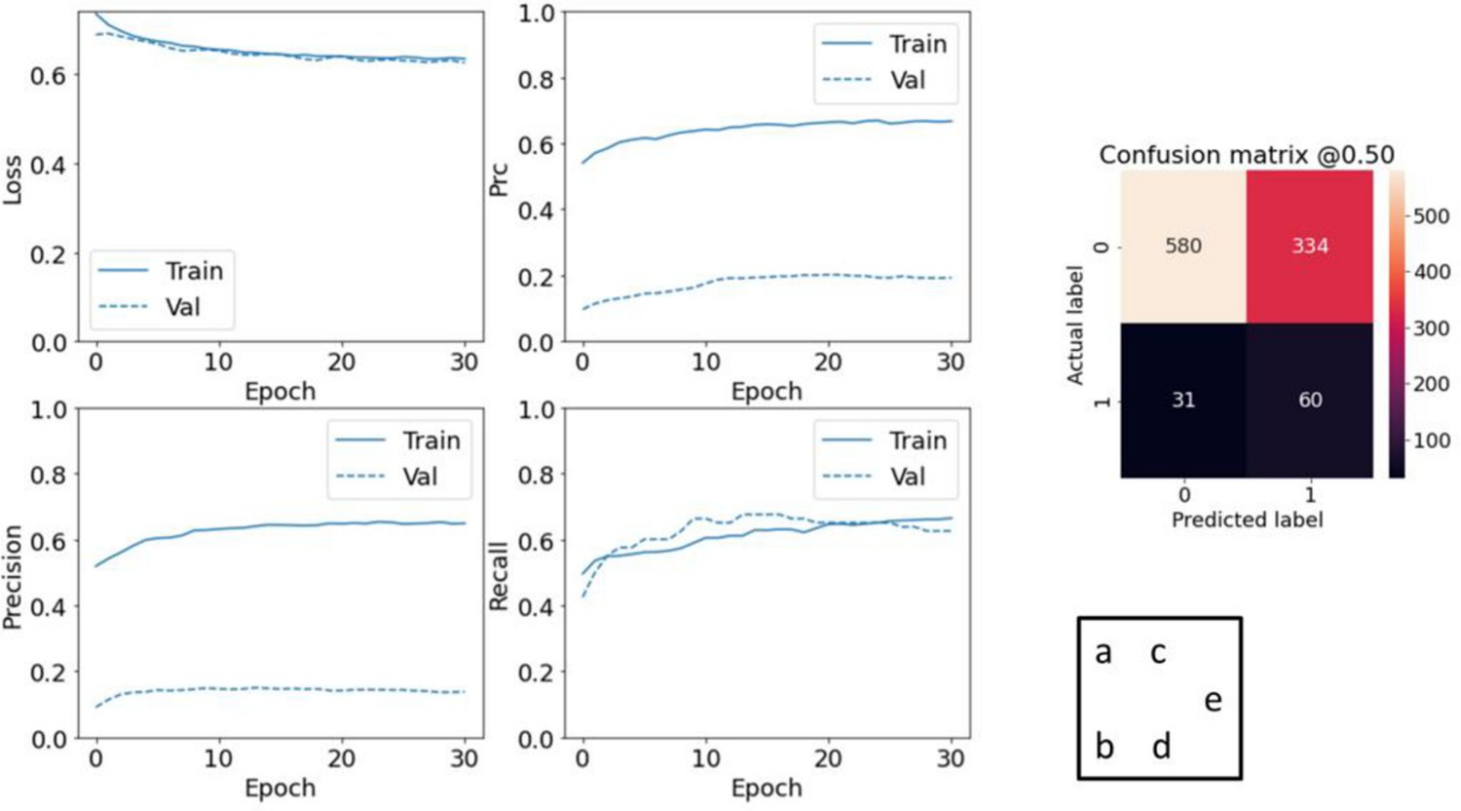
Evaluating resampled model performance. a-d) evaluate metrics; e) confusion matrix.

Although the class weighting method is more common to overcome imbalanced data, the oversampling method for this task has yielded better results. For this reason, in this study, by expressing the results related to class weighting, the oversampling method has been selected. This method resamples the dataset by oversampling the minority class. The database is manually balanced by selecting a random number of positive class instances. As the database is balanced by replicating the positive examples, the size of the database has increased. Because many positive examples have been repeated, the probability of over fitting increases rapidly. Early stopping has been used to prevent this problem. Fig 7 shows the various model evaluations along as well as the confusion matrix.

**Fig 8:**
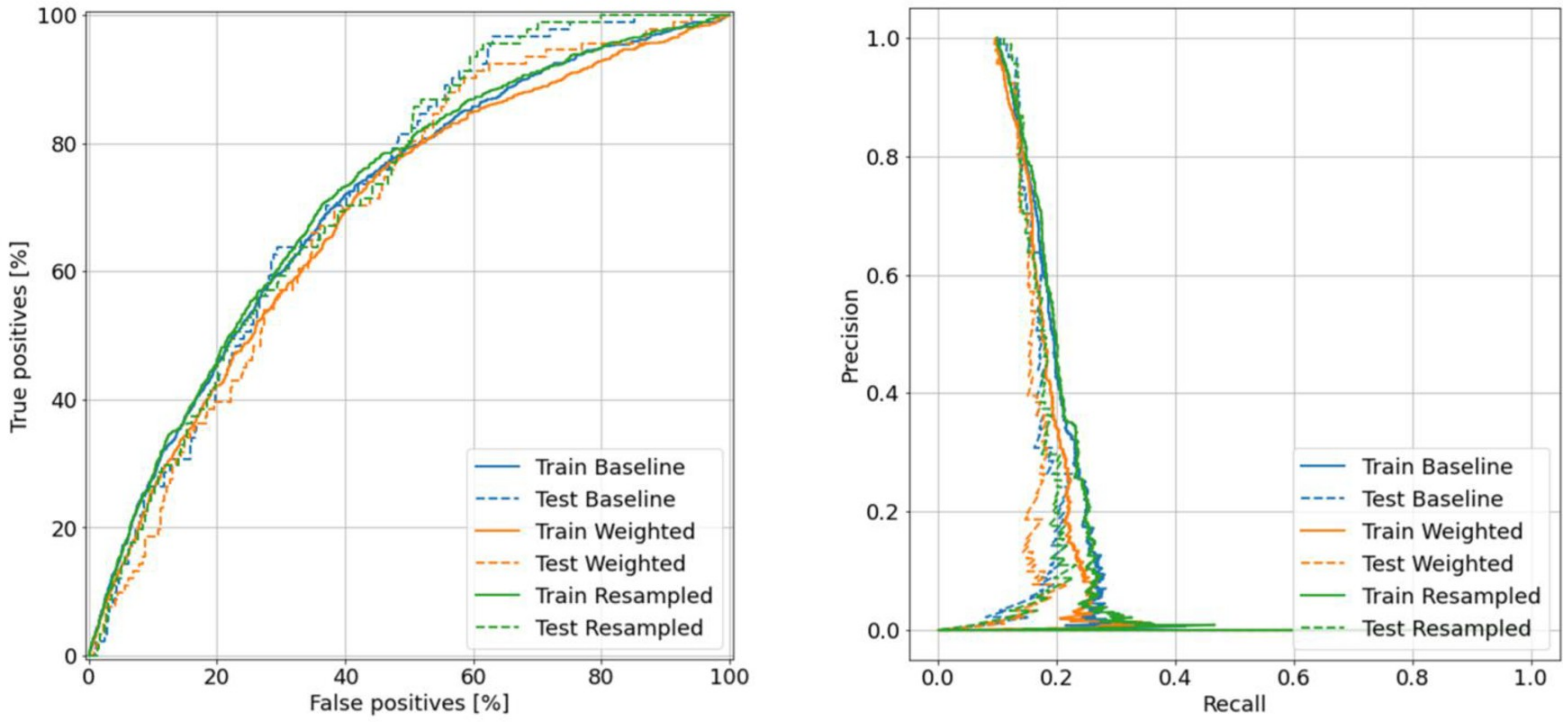
(left) AUC and (right) AUPRC of baseline, weighted, and resampled models.

Finally, Fig 8 shows the AUC and AUPRC diagrams of all three trained models. The AUC metric is the probability that the model will give a random positive sample a higher score than a random negative sample. AUPRC metric computes precision-recall pairs for different probability thresholds.

### 3.3. Feature importance

After cleaning the data, and using a 1-dimensional convolution Neural Network deep learning model for the prognosis of diabetes, the issue of interpreting of this model becomes important. Model interpretation allows us to find the importance of each feature in the model. For example, our prognosis model uses the following features for the risk of diabetes: Gender ID, BMI, Age (At Interview), Height (Cm), Weight (Kg), Waist Circumference, and Hip Circumference.

To determine the importance of a property, there are two common methods: drop column method and Permutation method. In the drop column method, a set of models is obtained by removing each of the features and training the main model on the remaining features as input. Using criteria such as C-index, each of these prognosis models is evaluated in a test set. The model with all features except gender has the highest c-index, followed by the model without Height (Cm) and the model without Weight (Kg). By examining the difference between the performance of various models with and without a particular attribute, the magnitude of its importance is determined. For example, the difference of C-index of the model in the presence or absence of the Waist Circumference feature is 0.07. Similarly, to examine the importance of Weight (Kg), we find that the difference of C-index between a model that uses this feature and one that does not include it is 0.06. So, we can understand that the importance of Waist Circumference is higher than the Weight (Kg) property. But, the challenge of this method is that we have to build several models. With two attributes, we have to make two additional models. With three attributes, we have to make three additional models. Therefore, we have to build additional models as much as the existing features and this is computationally very expensive. In addition, the similarity of different models that are made cannot be calculated.

In contrast, the Permutation method to solve the above challenge, instead of teaching several models to determine the importance of features, considers feature permutation. Once again, we have the same prognosis settings that were trained on all the features. Therefore, we have a set of tests from patients that we can evaluate the model and get the C-Index on the test data. In this method, we shuffle the values of one of the attribute columns, such as Height (Cm), and calculate the c-index value in this case on the test data. When a modified column is fed to the model, the performance of the model decreases. Similarly, we changed the Weight (Kg) property and transferred these values to the model again and obtained the model performance. The purpose of the Permutation method is to examine the drop of the model performance in the case where all feature columns have their true values as opposed to the case where a particular column is shuffled. Therefore, if a particular feature is important and its values are changed, we see a significant drop in performance. Here, the performance drop for Weight (Kg) is 0.2, while the performance drop for Height (Cm) is 0.1. So, it allows us to say that Weight (Kg) is more important than Height (Cm). The advantage of this method is that without re-training a model, it is used with different shuffled columns several times. Using the permutation method, the importance of the features taught by the deep learning model to predict diabetes is as follows: BMI, Waist Circumference, Age (At Interview), Hip Circumference, Weight (Kg), Height (Cm), and Gender ID.

### 3.4. Individual feature importance

In the previous section, we obtained the general importance of features for our deep learning model of the entire data set. But the important subject is that for a new patient A, which features are more important. Therefore, in this section, we get the importance of a feature to predict the disease of a particular person. With methods such as the permutation method, we generally obtained the significance of each property for the model. But, in this section, it is more important to understand a single prediction of a patient than to understand a model. For example, we want to know how important the Hip Circumference feature is for prediction of the deep learning model on patient A. Assume that patient A has the characteristics described in Table 2.

**Table 2:** An individual sample A against median of dataset features.

Sample	Gender ID	Age_At_Interview	Height Cm	Weight Kg	Waist Circumference	Hip Circumference	BMI
Patient A	male	60	159.7	121.2	133.0	150.0	47.52
Median (Total)	--	46	162.1	72.2	97.0	102.0	27.25
Median (T2DM)	--	52	160.2	72.8	99.5	102.0	28.09
Median (Not T2DM)	--	45	162.4	72.2	97.0	102.0	27.16

**Fig 9:**
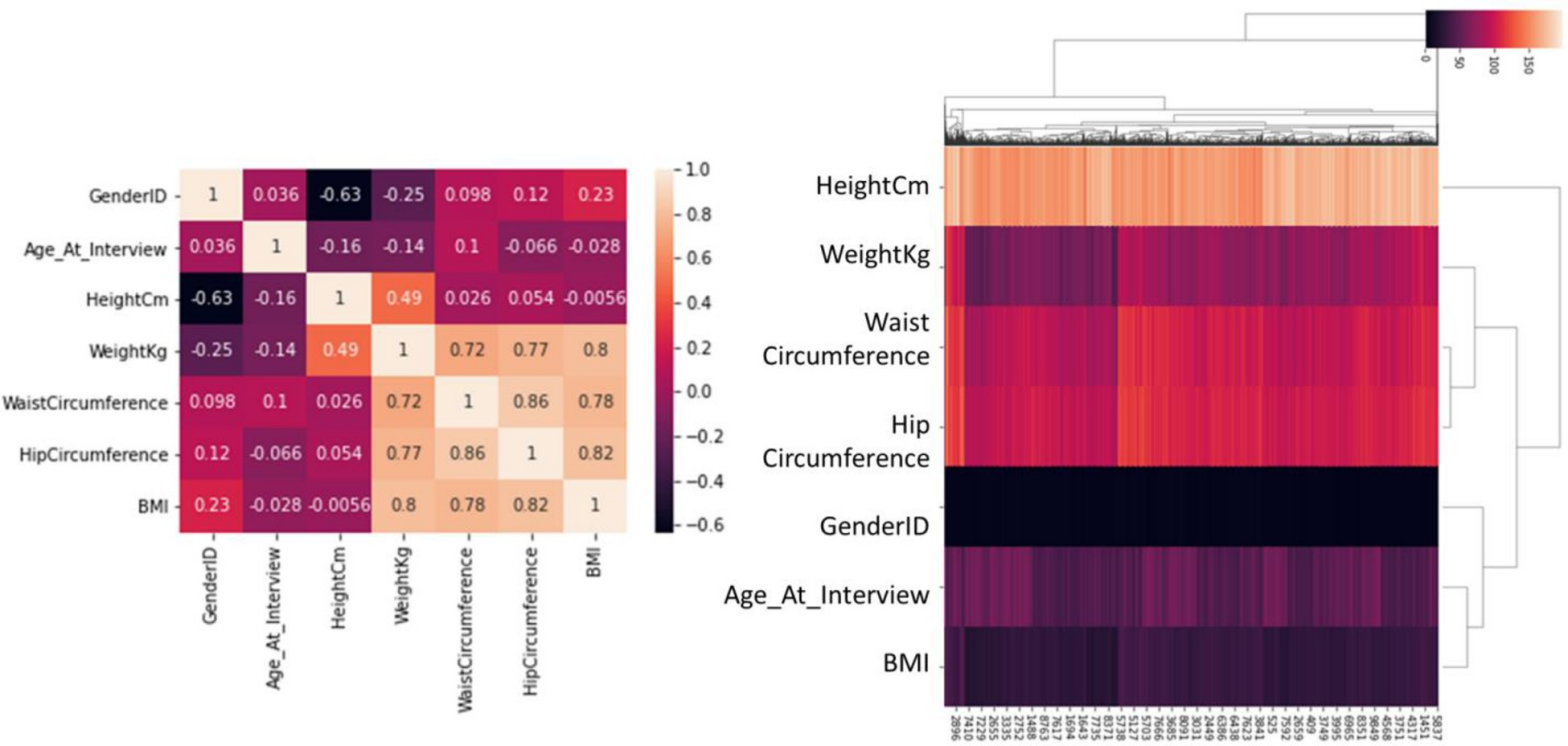
Heat-map of features with annotations and cluster-map of features.

As we can see in Table 2, patient A has a much higher hip circumference than the median of the statistical population. Although Waist Circumference is generally more important to patients, we expect that her/him Hip Circumference leads to further increase in her/him risk of diabetes. For this purpose, the deep learning model is trained on all the features of the data set and the amount of risk of having diabetes in patient A is predicted by the model. This value is called the base value for patient A. Then one of the feature columns is removed and a new model is taught. The amount of risk is predicted by the new model. The difference in the amount of risk between the two recent models indicates the importance of the presence of the feature that has been omitted. We did the same thing on the other features to get the importance of each feature. This method is exactly the same as the drop column method, except that instead of the model performance, we seek to survey the model output on patient A (model output on patient A indicates the amount of risk of diabetes in patient A that predicted by the model). However, this method cannot identify important features for patient A in the presence of correlated features. Because the correlated features enhance the effect of each other and the model incorrectly compensates the absence of a feature by its correlated feature, and as a result no reduction occurred in the model performance by removing one of the two correlated features. Fig 9 shows the correlation of the properties for the presented database:

Also, the right image of Fig 9 shows that the features of Waist Circumference and Hip Circumference are highly correlated. Table 2 shows both of these features are very high in patient A. So, as before, we should expect that the importance of both features is very high. If these two features were not correlated, the above method would correctly show the importance of each feature for patient A. But, if we consider the correlation between these two features, which is close to 0.86, the difference in the amount of risk for the presence and absence of Hip Circumference becomes small. Because at least one of these two measurements, Waist Circumference and Hip Circumference, is always in the feature set. Therefore, we always see a high risk prediction for patient A. Therefore, using the above method, the output difference of the model in the presence and absence of these features is small and we wrongly realize that the importance of each feature is very small, and we cannot recognize the importance of high waist circumference and high hip circumference for the patient A. To solve this problem, we have used Shapley values [19–21].

The values Shapley method can accurately indicate the importance of each features, even if there are correlated features. To get the importance of the Hip Circumference feature by values Shapley method, not only the complete set of features is considered, but also the whole set of features containing Hip Circumference is considered. Table 3 shows a few sample lines of features that the model compares. The number of these rows is **2^7−1^ = 64**, which is equal to the total number of cases in which Hip Circumference was present or not.

**Table 3:** All feature set with Hip Circumference on the left column vs. the corresponding feature set with Hip Circumference removed on the right column.

BMI, Waist Circumference, Weight (Kg), Height (Cm), Age (At Interview), Gender ID, and **Hip Circumference**.	vs.	BMI, Waist Circumference, Weight (Kg), Height (Cm), Age (At Interview), Gender ID.
Waist Circumference, Weight (Kg), Height (Cm), Age (At Interview), Gender ID, and **Hip Circumference**.	vs.	Waist Circumference, Weight (Kg), Height (Cm), Age (At Interview), Gender ID.
BMI, Weight (Kg), Height (Cm), Age (At Interview), Gender ID, and **Hip Circumference**.	vs.	BMI, Weight (Kg), Height (Cm), Age (At Interview), Gender ID.
BMI, Waist Circumference, Weight (Kg), Age (At Interview), Gender ID, and **Hip Circumference**.	vs.	BMI, Waist Circumference, Weight (Kg), Age (At Interview), Gender ID.
BMI, Waist Circumference, Weight (Kg), Height (Cm), Gender ID, and **Hip Circumference**.	vs.	BMI, Waist Circumference, Weight (Kg), Height (Cm), Gender ID.
BMI, Waist Circumference, Weight (Kg), Height (Cm), Age (At Interview), and **Hip Circumference**.	vs.	BMI, Waist Circumference, Weight (Kg), Height (Cm), Age (At Interview).
BMI, Height (Cm), Age (At Interview), Gender ID, and **Hip Circumference**.	vs.	BMI, Height (Cm), Age (At Interview), Gender ID.
BMI, Weight (Kg), Age (At Interview), Gender ID, and **Hip Circumference**.	vs.	BMI, Weight (Kg), Age (At Interview), Gender ID.
BMI, Weight (Kg), Height (Cm), Gender ID, and **Hip Circumference**.	vs.	BMI, Weight (Kg), Height (Cm), Gender ID.
BMI, Weight (Kg), Height (Cm), Age (At Interview), and **Hip Circumference**.	vs.	BMI, Weight (Kg), Height (Cm), Age (At Interview).
**…**	**…**	**…**
Gender ID, and **Hip Circumference**.	vs.	Gender ID
**Hip Circumference**.	vs.	{}

**Fig 10:**
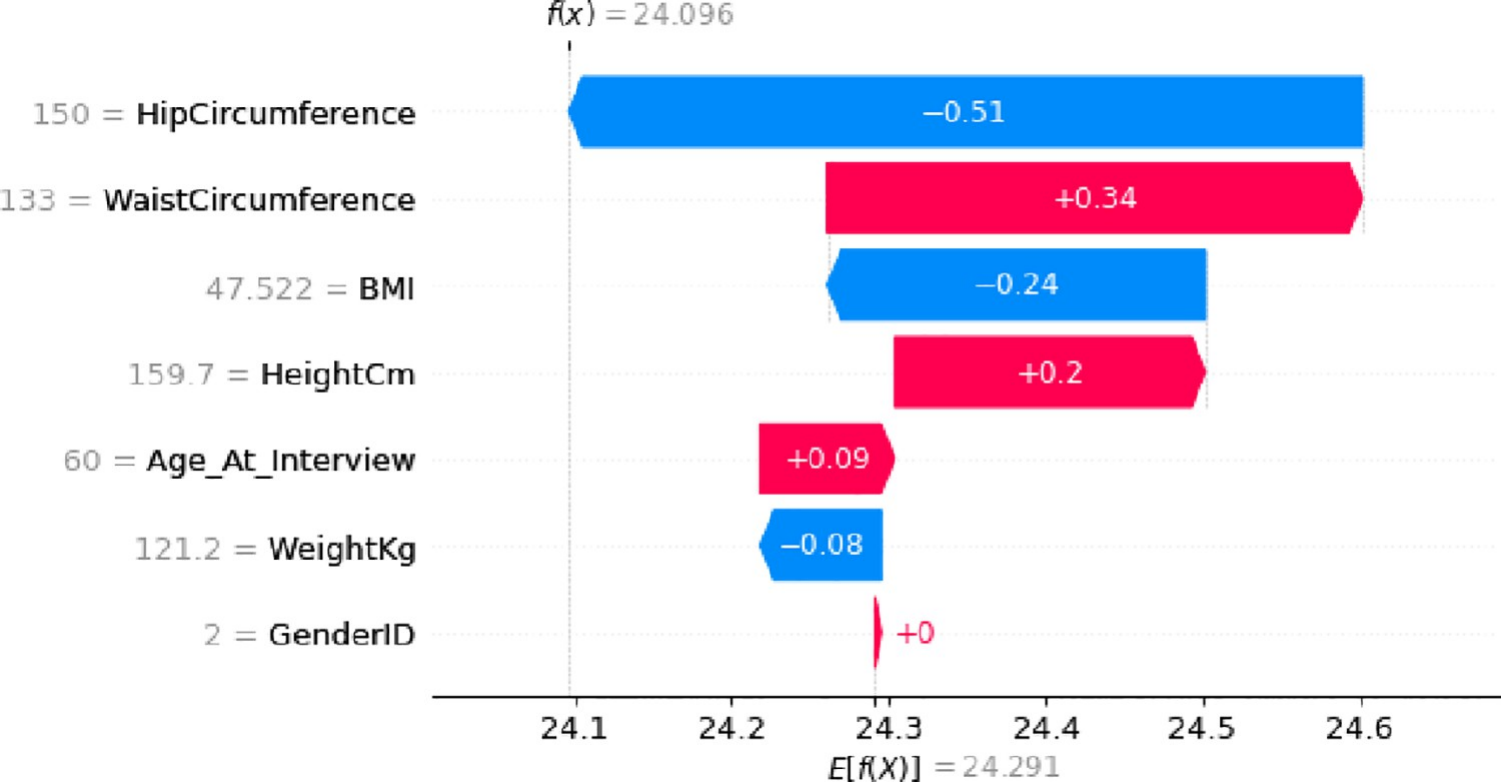
Feature contribution for predicting the risk of patient A.

The left side of Table 3, all the feature sets includes Hip Circumference and the right side includes the corresponding feature set with removed Hip Circumference. The last item here is an empty set, which is a set that has no features. Therefore, we have trained 64 * 2 = 128 models that predict patient A with this subset of different features. From these cases, 64 cases contain Hip Circumference and the other 64 items do not contain it. The special case here is the empty set. This row is very important because it represents the ratio of the number of data in each class. By considering the difference between prediction with and without feature, we have calculated the importance of Hip Circumference for each of these features subsets. So, here we obtained 64 numbers that give us the importance of Hip Circumference for predicting patient A with each of these features sets. These 64 numbers must be combined to obtain a single number that indicates the importance of Hip Circumference for patient A. For combining importance, we have created a set of features at a time so that our feature set is empty at first. A feature was randomly selected such as Height (Cm), and then another feature was randomly selected without placement, such as Gender ID, and random selection without placement was continued until we reached the Hip Circumference feature. Here Hip Circumference was the third random selection. We obtained the prediction difference of the first model, which includes all three of the above features, and the second model, which does not include Hip Circumference. We do the same calculation of the prediction difference for all feature modes, which is 7!. Finally, the importance of the Hip Circumference feature is the average of all 7! numbers that we have obtained. This number is considered as the Shapley value of the Hip Circumference feature for patient A. Similarly, Shapley values have been calculated for all features. The contribution of Hip Circumference and Waist Circumference seems to be much higher than other features of patient A. Eventually, the maximum risk of patient A for having diabetes is 24,096, as shown in Fig 10. This figure shows the effect of risk for patient A according to the importance of features so that the effect of features starts from the base value of the model output (the average of output of the model from the training data set i.e. 24.291). Features that raise the prediction have been shown in red color, and features that reduce the prediction are shown in blue one.

**Fig 11:**
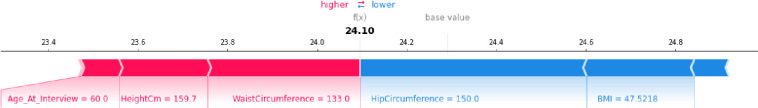
Force plot of feature contribution for predicting the diabetes risk of patient A.

Fig 11 shows the force plot of the above explanation. In this figure, the risk output value is 24.1, which is less than the base value = 24.291. As can be seen in the force plot, the greatest effect is related to the longest feature, namely Hip Circumference, which its blue color indicates its negative effect (-0.51). After that, waist circumference has the most effect (+0.34). Therefore, although BMI was the most important feature of the entire training data set, Hip Circumference is the most important feature for patient A. According to Shapley value, the risk of developing diabetes in patient A is 24,096, while the model singly had a risk of 24.291% for this patient.

**Fig 12:**
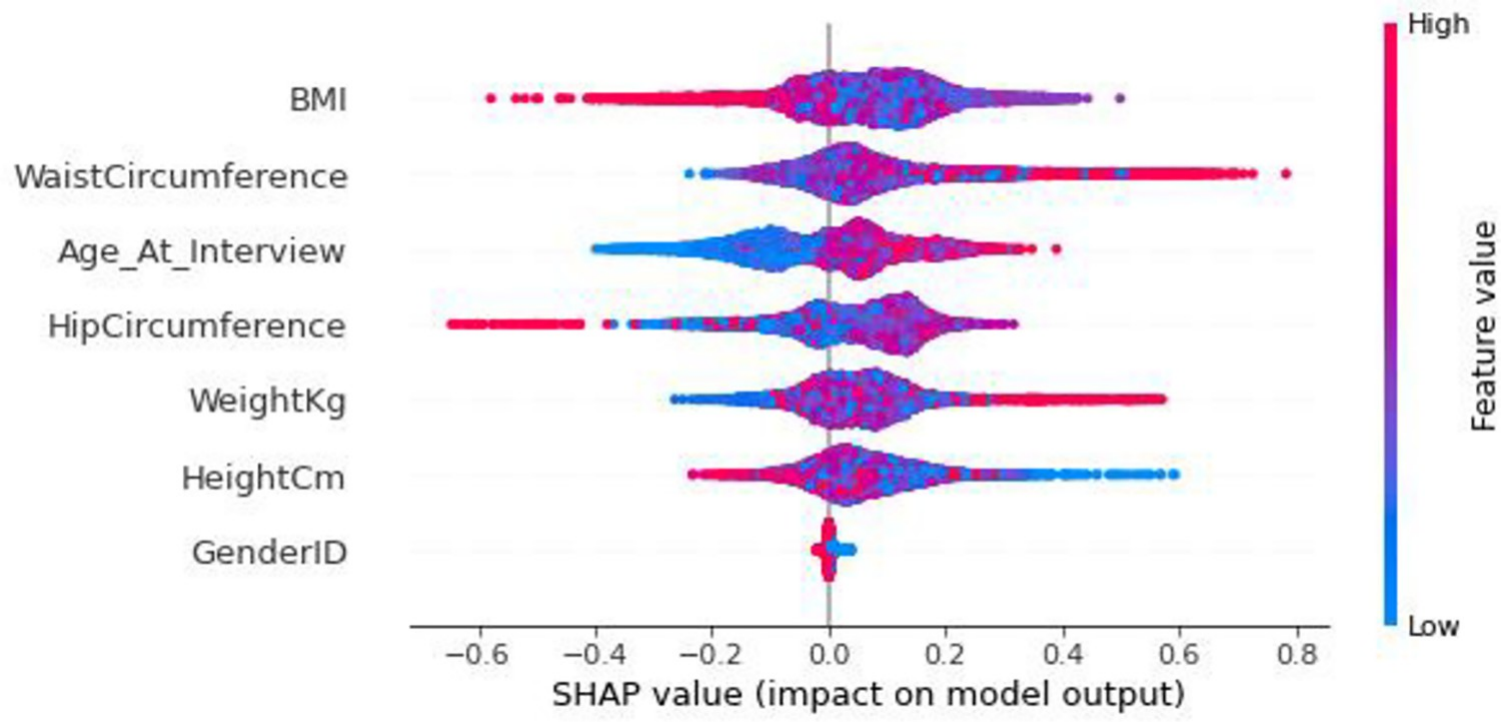
Summary plot of Shapley values for all patients, aim to predict the diabetes risk.

We have calculated the Shapley values for all patients in a data set. Using the summary plot in Fig 12, we visualize the Shapley values distribution for each feature. Each point in this diagram represents a characteristic of a patient. The set of points in this chart represents the distribution of Shapley Values for all patients.

In this chart we see that the Waist Circumference feature has positive values for many patients in this dataset, and the Hip Circumference feature has negative values for many patients. Here the red color means the high value of the feature. For example, when a point for Hip Circumference is red, it means that this feature has a high value for the considered patient. Similarly, a blue dot represents a small value of feature. For example, blue dot for Age (At Interview) means that the considered patient’s age is young. On the other hand, as much as any point on the chart tends to the right side of the figure, the risk of developing diabetes in the patient increases. So here, increasing the patient’s waist circumference increases the risk of diabetes. Because the right side of the chart has significant red values of the waist circumference feature.

**Fig 13:**
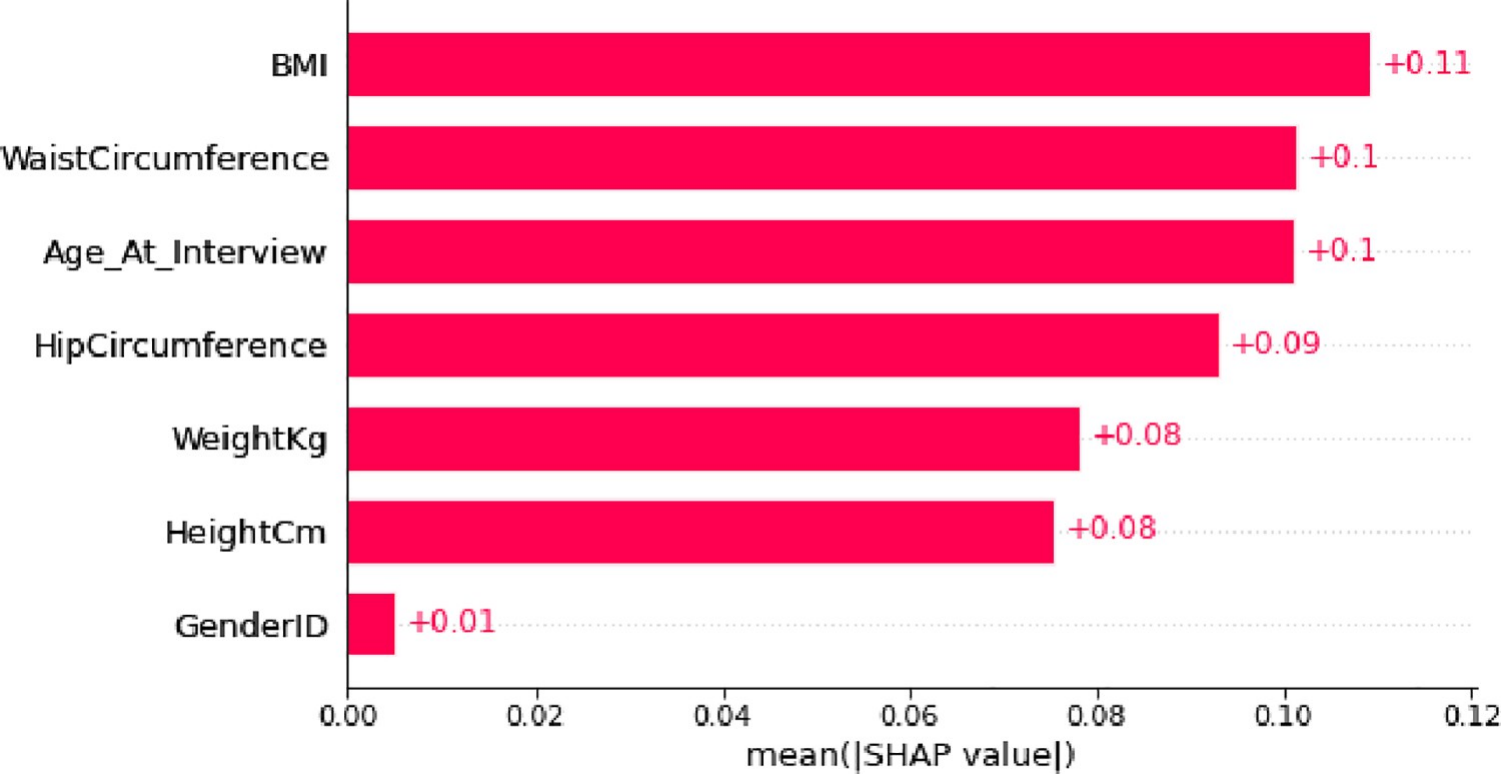
Bar summary plot for the absolute value of the Shapley values, to show the feature importance overall.

In Fig 13, we show the overall importance of a feature by bar summary plot. These values has been calculated by considering the absolute value of the Shapley values for all patients and then taking the average of those Shapley values for each feature. Thus, the average absolute value of Shapley values has been obtained by this method, and from Shapley individual values, we have reached the global significance in a population in this graph. We can say that BMI is the most important feature in the general and then Waist Circumference is very important.

**Fig 14:**
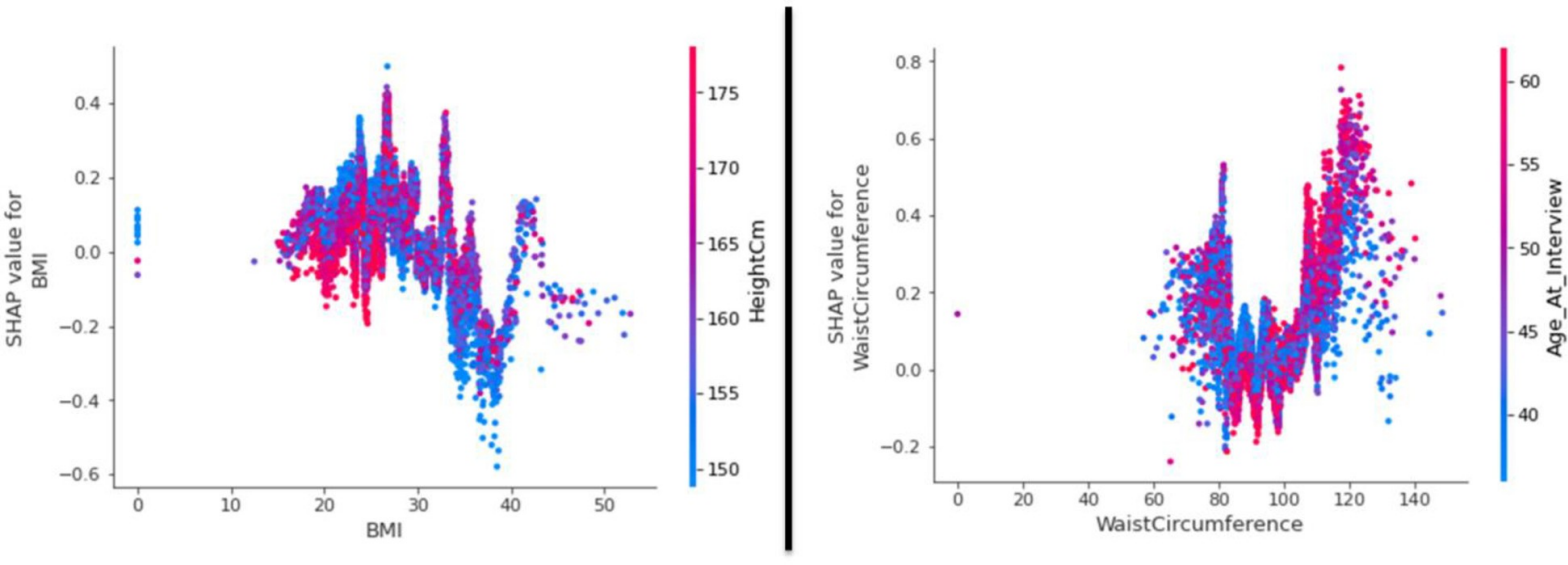
Influence of BMI and waist circumference features on model output.

Fig 14 shows how these two important features affect output. To do this, the shapely value of these features has been plotted for all data set examples. For example, the diagram on the right side shows the contribution of the Waist Circumference feature to change the model output. In other words, the change in the prediction of the risk of diabetes of the model is related to a change in Waist Circumference. Vertical dispersion in a single value of the horizontal axis represents interaction effects with other features.

**Fig 15:**
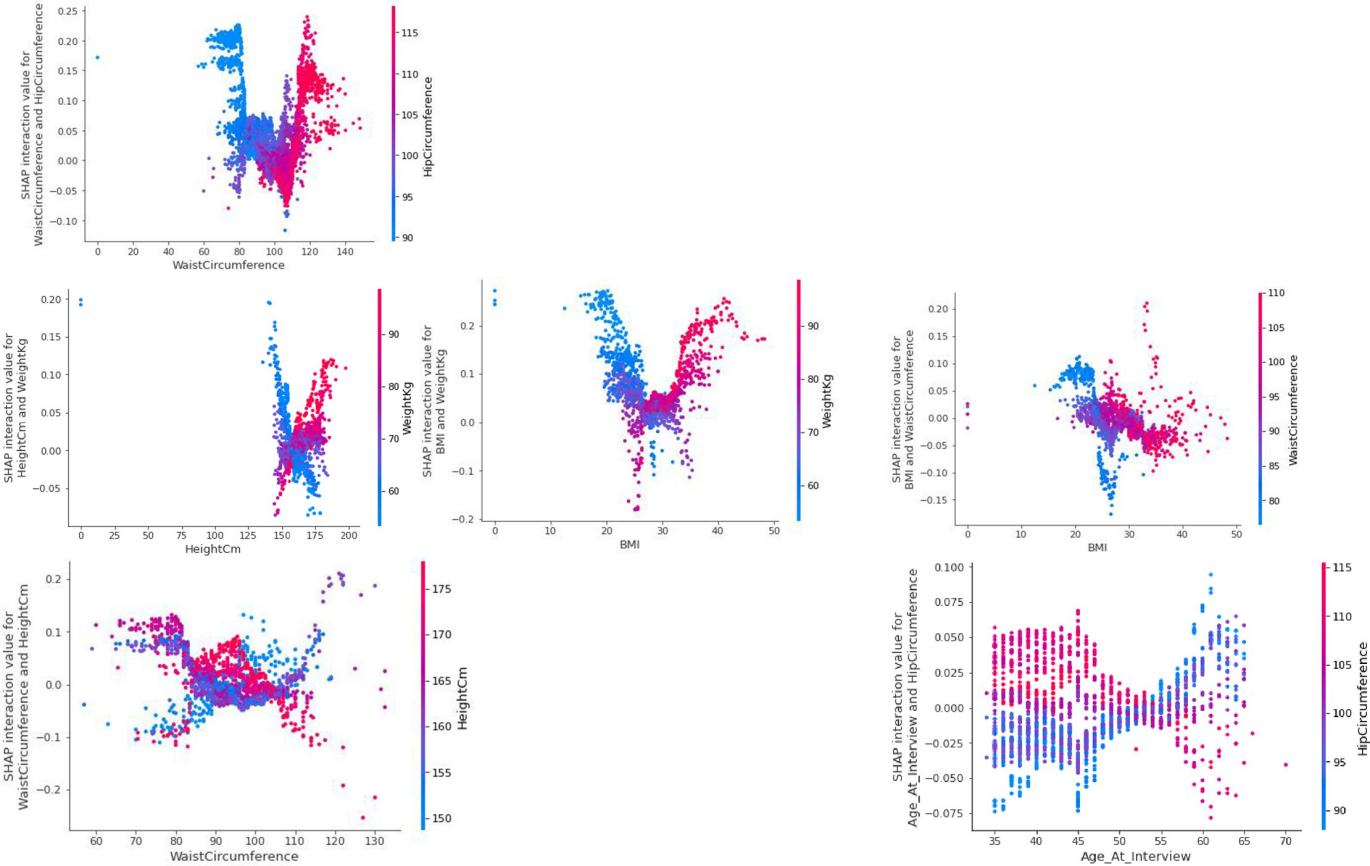
Shapley interaction values for different socio-demographic features.

Fig 15 shows different dependence plots of socio-demographic features between two important characteristics at risk of diabetes. For example, the top left plot shows the Shapley interaction values for Waist Circumference and Hip Circumference. As can be seen, in most patients in this set, when the waist circumference is high, the risk of disease is so high (red color) that hip circumference has very little effect on the model output. In contrast, in most patients with low Waist Circumference, patients with a lower ratio of $\frac{Waist\;Circumference}{Hip\;Circumference}$, had the lowest risk of developing diabetes.

**Fig 16:**
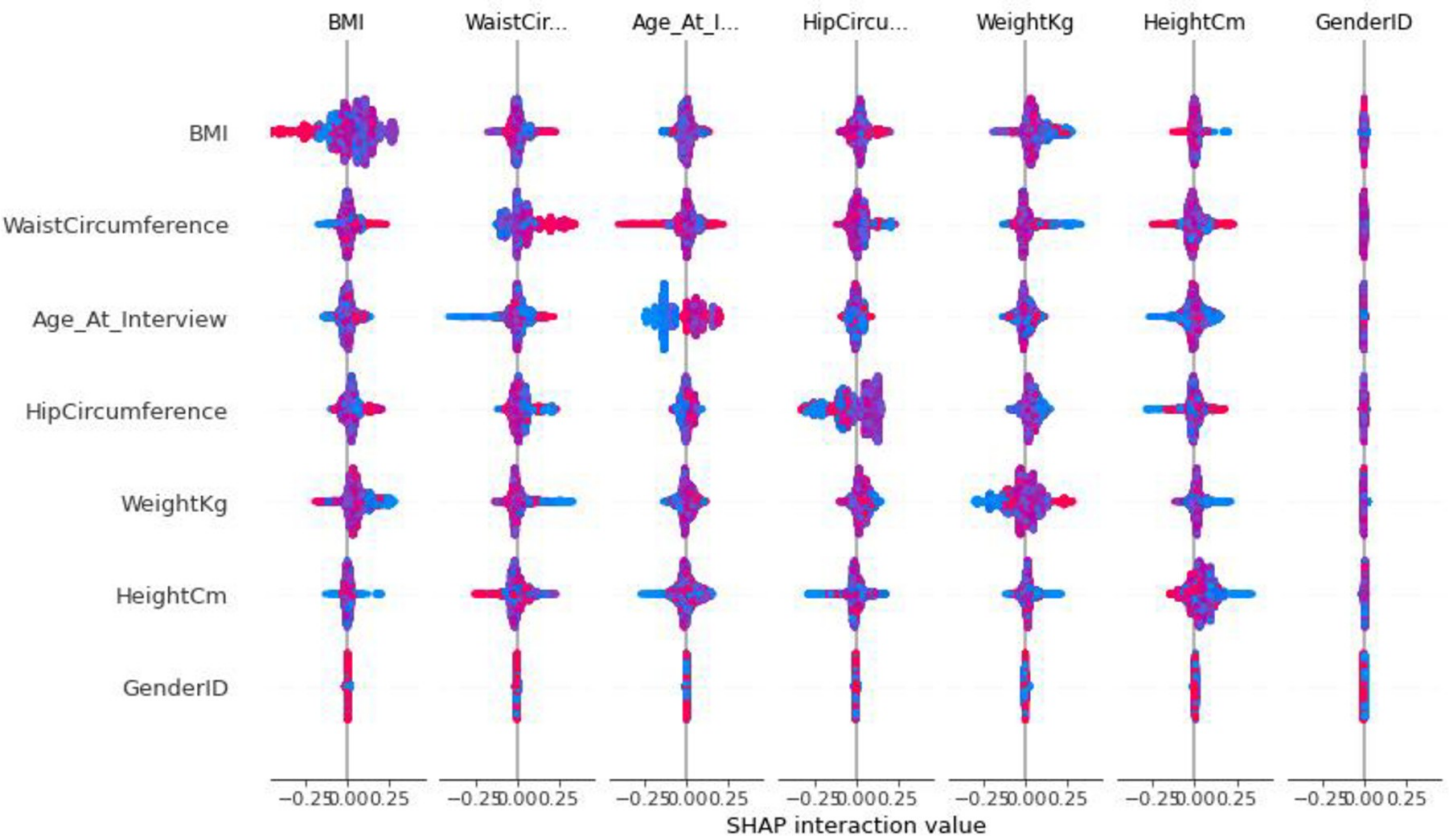
Shapley interact value for all features.

Finally, Fig 16 shows a summary of the Shapley interaction value diagram for all features on all the data set predicted by the model.

## 4. Discussion

In this article, we investigate the anthropometric characteristics of patients with chronic diseases (diabetes, hypertension, cardiovascular disease, heart attacks and strokes) and find the factors affecting these diseases and the extent of the impact of each to apply the necessary planning for prevention.

Using deep learning, this study uses a model to diagnose the risk of T2DM. Positive class data were much less than samples with negative class. Hence, we have the problem of Imbalanced data. To overcome this problem, two new models were created so that the classes were weighted and data became oversampling. To solve the over fitting problem, dropout layer and early-stopping technique were used. Conventional metrics were considered to evaluate these models. Accuracy metric was not a good evaluation criterion for this task. Because a simple model that negatively labels all new people has an accuracy = 90.36. In this task, the diagnosis of T2DM patients is more important. In other words, the goal is to reduce the error in which a random sample has a positive class and the model incorrectly recognizes it as a negative class (False Negative). For this reason, Precision and Recall metrics have been used to evaluate the model. AUC and AUPRC criteria were used in this study to consider both metrics simultaneously. Finally, the model that used the oversampling method had better results by evaluating metrics.

After fixing the model and training the deep neural network with this model, the importance of features was explained. This is important because the risk of getting the disease in various persons has different dependence on the characteristics. For example, a young person with a high BMI, BMI is a more important criterion than his age in the risk of T2DM. Conversely, in an older person with a moderate BMI, age feature is more important than BMI. This is called individual feature importance, and in this study, Shapley values were used to personalize the model on each new instance. By showing a hypothetical case, the risk of T2DM in that was determined by extracting the probabilistic value from the model to predict T2DM in this subject. Then, each feature shifted the risk of the disease according to its importance in that person. Finally, the sequence used in this study has been explored the risk of developing T2DM for a particular individual with personality capability.

The available information from the target community can provide an opportunity to take precaution action in order to control some of chronic diseases and to prevent the cost of the treatment. Therefore, the aim of this study was to investigate the anthropometric characteristics of patients with chronic diseases (diabetes, hypertension, cardiovascular disease, heart attack and stroke) and to find the factors affecting these diseases and the extent of their impact to perform the necessary plans.

This paper focused on diagnosis and prognosis of chronic diseases using anthropometric characteristics on the Ravansar county cohort study. The diseases include diabetes, myocardial ischemia, myocardial infarction (MI), stroke, renal Failure, fatty liver, rheumatic disease, multiple sclerosis (MS), pregnancy hypertension, and pregnancy diabetes. The goal was to find the factors affecting these diseases and the extent of the impact of each to make the necessary planning. After data creation, the T2DM disease diagnosed during the pipeline of data preprocessing, handling imbalanced data, three different deep learning models, true evaluation method, Feature importance and individual feature importance, as a case study. Through the results, the pipeline demonstrated competence in improving the diagnosis and prognosis the risk of T2DM with personalization capability. The personalization capability helps us to better prognosis the disease for individuals.

Some anthropometric indicators have a significant relationship with risk factors of chronic diseases. So, continuous evaluations, lifestyle changes and increasing the level of awareness to control, prevent and adjust the indicators are suggested.
